# Esthesioneuroblastoma metastasis to the breast: A case report and review of the literature

**DOI:** 10.3892/ol.2014.2403

**Published:** 2014-08-01

**Authors:** NOPPADOL LARBCHAROENSUB, WASANA KANOKSIL, WICHIT CHEEWARUANGROJ, CHOLATIP WIRATKAPUN, CHOMPORN SITATHANEE, EKAPHOP SIRACHAINAN

**Affiliations:** 1 Department of Pathology, Faculty of Medicine, Ramathibodi Hospital, Mahidol University, Ratchathewi, Bangkok 10400, Thailand; 2 Department of Otolaryngology, Faculty of Medicine, Ramathibodi Hospital, Mahidol University, Ratchathewi, Bangkok 10400, Thailand; 3 Department of Radiology, Faculty of Medicine, Ramathibodi Hospital, Mahidol University, Ratchathewi, Bangkok 10400, Thailand; 4 Department of Medicine, Faculty of Medicine, Ramathibodi Hospital, Mahidol University, Ratchathewi, Bangkok 10400, Thailand

**Keywords:** esthesioneutroblastoma, olfactory neuroblastoma, metastasis, breast

## Abstract

Metastasis to the breast from an extramammary malignant neoplasm, including esthesioneuroblastoma, is uncommon. The present study describes a rare case of sinonasal esthesioneuroblastoma, Hyams’ histologic grade 2, Kadish’s stage C, T4N0M0, in a 30-year-old female. The patient underwent a radical ethmoidectomy with external beam radiotherapy, followed by chemotherapy including five cycles of cisplatin and etoposide. One year after the initial diagnosis, the patient presented to the hospital with the chief complaint of a rapidly enlarging lump in the right breast. A fine needle aspiration was performed and immunocytochemistry revealed a metastatic esthesioneuroblastoma. The patient received palliative chemotherapy and radiotherapy; however, the patient developed a local recurrence with systemic metastasis and succumbed to the disease seven months later.

## Introduction

Esthesioneuroblastoma (ENB) is an uncommon malignant neuroectodermal tumor originating from the olfactory membrane of the sinonasal tract (1). The various appellations include olfactory neuroblastoma, esthesioneurocytoma, esthesioneuroepithelioma and olfactory placode tumor (1,2). The incidence has been reported at 0.4 per million (1). Complete surgical eradication including craniofacial resection followed by radiotherapy is the cornerstone of treatment. Chemotherapy is employed in patients with advance locoregional, local recurrent or metastatic disease and is used as adjuvant therapy in high grade, Kadish’s stage C, ENB (1,3). An endoscopic microsurgical technique has gained wide acceptance for a novel therapeutic approach of ENB (4). The overall five-year survival rate is 78% (1). A regional sinonasal involvement is characteristic, but metastasis is uncommon. The most common metastatic sites are the cervical lymph nodes, lungs and bone (1,2). Metastasis in the breast from extramammary malignant neoplasm is uncommon (5). The present study reports of a rare case of breast metastasis in a 30-year-old female with ENB (Kadish’s stage C). The patient underwent a radical ethmoidectomy with radiotherapy followed by chemotherapy including platinum-based protocols.

## Case report

### Clinical summary

**Figure 1:**
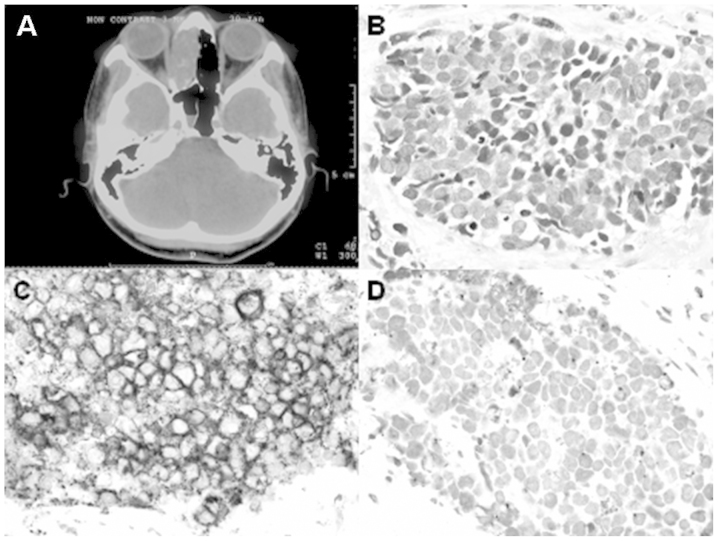
Computed tomography shows (A) a poorly-defined mass in the nasal cavity. (B) Histopathology reveals groups of polygonal tumor cells (hematoxylin and eosin stain). Immunohistochemistry shows (C) positive staining for CD56 and (D) negative staining for cytokeratin. Magnification, ×400.

**Figure 2:**
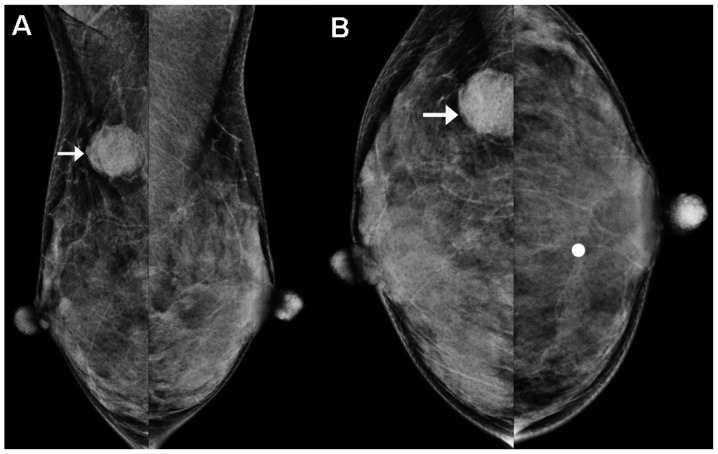
Mammogram on the (A) mediolateral oblique and (B) craniocaudal view show a hyperdense mass (arrows) with a circumscribed border at the upper-outer quadrant of the right breast.

**Figure 3:**
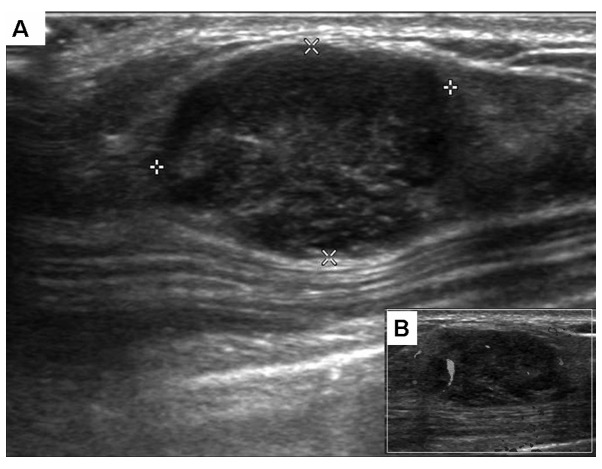
(A) Ultrasound shows a solid mass with heterogeneous internal echogenicity and a gently lobulated border. (B) Color Doppler ultrasound reveals an intralesional vascular flow.

A 31-year-old Thai female with known ENB, Hyams’ histologic grade 2, Kadish’s stage C, T4N0M0, presented with a rapidly enlarging lump of the right breast. Fourteen months previously, the patient was referred to the Faculty of Medicine, Ramathibodi Hospital (Bangkok, Thailand) due to nasal congestion. A radical ethmoidectomy was performed and the pathology was shown to be ENB with multiple foci of lymphovascular space invasion (Fig. 1). The patient received 5,000 cGy of external beam radiotherapy, followed by postoperative chemotherapy including five cycles of cisplatin and etoposide. The patient was disease-free for seven months prior to presenting at the hospital four days prior to this admission with a rapidly enlarging lump in her right breast and a cervical lymphadenopathy. A physical examination revealed a palpable, 2.5-cm, firm and movable mass in the right breast. A digital mammography showed a hyperdense mass with circumscribed border in the background of extremely dense fibroglandular mammary tissue at the upper outer quadrant of the right breast (Fig. 2). No associated microcalcification was identified. Ultrasonography showed a solid mass with heterogenous echogenicity and a gently lobulated border measuring 2.2×1.4×2.6 cm. A posterior acoustic enhancement was observed (Fig. 3A). Intralesional blood vessels were observed on the color Doppler ultrasound (Fig. 3B). No change of the overlying skin or axillary lymphadenopathy was observed. The patient underwent a fine needle aspiration (FNA), and a systemic work-up revealed pulmonary and mediastinal nodal metastases. Subsequently, the patient received two courses of chemotherapy, including vincristine, doxorubicin and cyclophosphamide and 300 cGy of palliative radiation to the chest. The patient then refused further treatment. Local recurrence and systemic vertebral, pulmonary and nodal metastases were detected, and the patient developed septicemia. Hemoculture grew *Candida albicans*. The patient succumbed to the disease seven months after the diagnosis of metastatic ENB to the right breast. No autopsy was performed.

### Cytopathological findings

**Figure 4:**
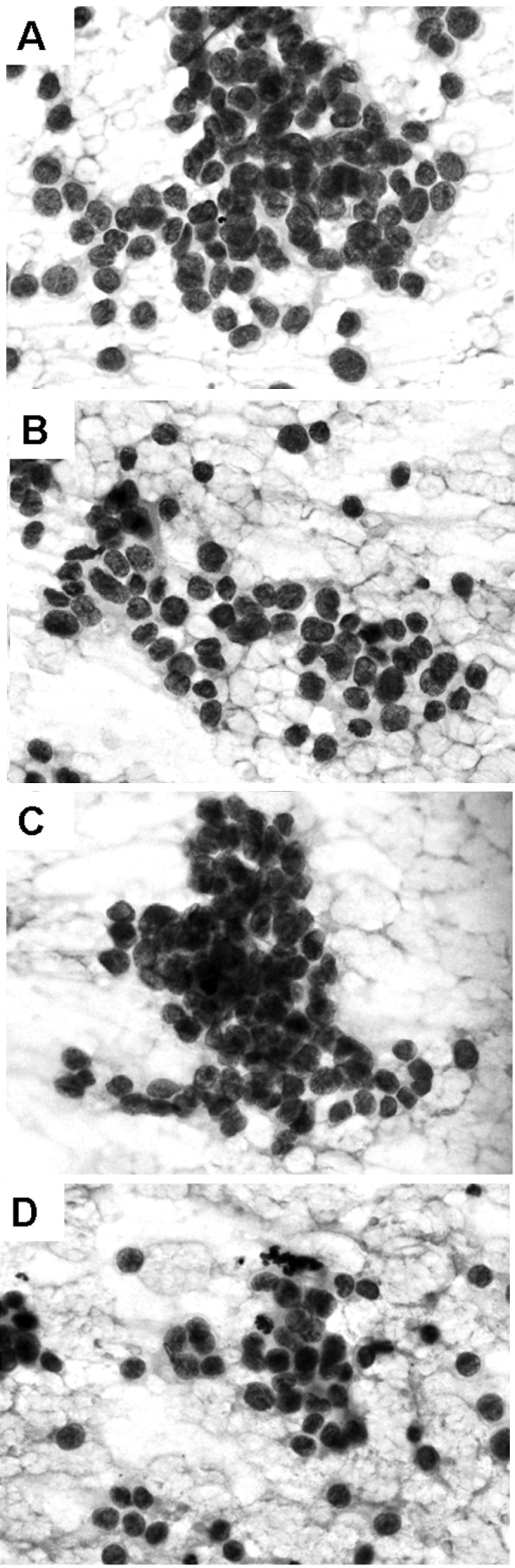
Cytology of the metastatic esthesioneuroblastoma in the breast shows areas of single/loose groups of cells with round to oval nuclei, coarsely granular chromatin, inconspicuous nucleoli and a focal area with a rosette formation (magnification, ×400).

FNA of the breast mass revealed neuroendocrine cells with slightly pleomorphic hyperchromatic nuclei, salt and pepper chromatin, indistinct nucleoli and scant cytoplasm (Fig. 4). Immunocytochemically, the neuroendocrine cells were positive for CD56, and negative for cytokeratin and gross cystic disease fluid protein-15. Morphologically and immunocytochemically, these characteristics were almost identical to the tumor observed in the sinonasal ethmoidectomy specimen.

## Discussion

The overall incidence of extramammary metastasis to the breast is 0.2–1.3% of all malignant breast tumors (5). Patients with non-Hodgkin lymphoma, melanoma, and carcinoma of the lung, stomach, ovary, kidney and colorectum in adults and children with rhabdomyosarcoma, have a higher risk of metastasis to the breast (5). Table I compares the present rare case of ENB metastasis with two reported cases of ENB metastasis to the breast that have been previously described in the English literature (Table I) (6,7). The patients had an average age of 20.3 years with a range of 13 to 30 years at the first time of diagnosis. All cases were at Kadish’s stage C. Mammary metastasis was reported to appear during the course of the treatment two years following the ENB diagnosis. The patients typically presented with a palpable breast mass, generally well-circumscribed and rapidly growing. All cases presented as a right breast lump and all succumbed following the diagnosis of esthesioneuroblastoma metastasis to the breast.

The mammographic and ultrasonographic appearances of the extramammary neoplasm metastasis to the breast may mimic benign mammary neoplasm and primary malignancy including carcinoma with medullary features. Metastatic tumors to the breast have three classic radiological patterns, consisting of: i) solitary tumor with a well-circumscribed border (as was exhibited in the presented case); ii) multiple diffuse and bilateral involvement; and iii) diffuse skin and trabecular thickening (8–12). Unlike the classical appearance of primary invasive mammary carcinoma, particularly ductal subtype, a speculated border is typically observed, since there is little or no desmoplastic reaction. Microcalcification is not a typical feature of metastatic tumors, with the exception of the previously reported case of metastatic ovarian serous carcinoma with psammoma bodies (9). Awareness of the patient history of extramammary cancer is essential in order to give an accurate diagnosis. Mammographic and ultrasonographic imaging studies may facilitate the diagnosis, but a full diagnosis should be established after a cytohistopathologic biopsy is performed.

It is important to distinguish between a primary mammary neoplasm and a metastasis in the breast, as well as to consider the possibly of a metastasis from an extramammary malignancy. This is particularly crucial with the increasing use of fine needle aspiration. The combination of cytology and immunocytochemistry is useful in separating metastasis from a primary malignancy (13). Identification and confirmation of the primary tumor is important to facilitate treatment. The treatment of a metastatic tumor is usually expectant and directed at treating the primary tumor. A mastectomy is generally not performed for metastatic tumors in the breast; however, wide excision may be performed to obtain local control of bulky, ulcerated, bleeding, necrotic or otherwise symptomatic lesions. The overall prognosis is dependent on the histopathology, tumor grade and tumor stage of the primary malignancy. Therefore, it is noteworthy to consider the possibility of an ENB metastasis to the breast when diagnosing a mass lesion of breast. Early diagnosis and prompt medical treatment are essential.

ENB is a rare neoplasm originating from the olfactory membrane of the sinonasal tract and has a high incidence of local recurrence. A systemic metastasis is uncommon. The present case report highlights the unusual site of a metastasis from ENB to the breast. ENB metastasis to the breast commonly presents in young adults, occurs with ENB with Kadish’s stage C, favors the right breast, is accompanied by systemic metastasis, exhibits rapid growth of mammary lump, has a poor response to chemotherapy and/or radiotherapy, carries a short disease free survival and overall survival, and portends a poor prognosis.

## Figures and Tables

**Table I:** Summary of cases of esthesioneuroblastoma metastasis to the breast.

Author (ref.)	Gender	Age at ENB diagnosis (years)	Symptoms	Kadish’s stage	Radiotherapy (cGy)	Chemotherapy	Interval between ENB and breast metastasis (months)	Breast metastasis			Systemic metastasis	Survival

								Side	Site	Size (mm)		
Shetty *et al* (4)	F	13	Left facial pain, nasal obstruction, epiphora, anosmia and epistaxis	C	5500	Cyclophosphamide and vincristine	During the course of radiotherapy	Right	NP	NP	Bone marrow and right ovary	Died during the course of radiotherapy
Mrad *et al* (5)	F	18	NP	C	NP	NP	24	Right	Lower inner quadrant	30	Vertebra	Died
Present case	F	30	Nasal stuffiness	C	5000	Cisplatin and etoposide	7	Right	Upper outer quadrant	22	Vertebra, lung and lymph node	Died 14 months after diagnosis

ENB, esthesioneuroblastoma; F, female; NP, Not performed.
